# Measuring cerebral glucose metabolism by chemical exchange-sensitive spin-lock (CESL) MRI of 2-deoxy-D-glucose in rodents

**DOI:** 10.1371/journal.pone.0346046

**Published:** 2026-03-27

**Authors:** Philipp Boehm-Sturm, Patrick Schuenke, Marco Foddis, Susanne Mueller, Stefan P. Koch, Daniel J. Beard, Paul Holloway, Amin Mottahedin, Leif Schröder, Alastair M. Buchan, Philipp Mergenthaler

**Affiliations:** 1 Charité-Universitätsmedizin Berlin, Center for Stroke Research Berlin, Berlin, Germany; 2 Charité-Universitätsmedizin Berlin, Experimental Imaging at the Charité for 3R (EPIC3R), Charité 3R – Replace | Reduce | Refine, Berlin, Germany; 3 Charité-Universitätsmedizin Berlin, Department of Neurology with Experimental Neurology, Berlin, Germany; 4 Charité-Universitätsmedizin Berlin, Department of Radiology, Berlin, Germany; 5 Physikalisch-Technische Bundesanstalt (PTB), Braunschweig and Berlin, Germany; 6 The University of Newcastle, School of Biomedical Sciences and Pharmacy, Newcastle, Australia; 7 Radcliffe Department of Medicine, University of Oxford, Oxford, United Kingdom; 8 Consortium International pour la Recherche Circadienne sur l’AVC (CIRCA); 9 Nuffield Department of Clinical Neurosciences, University of Oxford, Oxford, United Kingdom; 10 German Cancer Research Center, Translational Molecular Imaging, Heidelberg, Germany; 11 Department for Physics and Astronomy, Ruprecht Karls University Heidelberg, Heidelberg, Germany; 12 German Cancer Consortium (DKTK), Heidelberg, Germany; Georgetown University Medical Centre, UNITED STATES OF AMERICA

## Abstract

Magnetic resonance imaging (MRI) of glucose metabolism shows significant potential for identifying disease biomarkers and monitoring therapeutic responses in neurological conditions. Here, we present a protocol utilizing chemical exchange-sensitive spin-lock (CESL) MRI with the glucose analogue 2-deoxy-D-glucose (2DG) in the rat brain. We employed this method to characterize metabolic changes in ischemic tissue in a rat model of stroke. However, the utility of the technique is not limited to stroke and may be adapted to other disease models with minimal modifications. Previous research has demonstrated that CESL MRI is sensitive to various glucose analogs, including regular D-glucose, which is suitable for human application. Consequently, our protocol provides a foundation for a wide range of future applications in both basic and translational research, with potential utility in animal models and, eventually, human studies.

## Introduction

Cerebral glucose metabolism is critical to sustain brain function, as emphasized by the fact that the brain is the main consumer of glucose-derived energy in mammals [1,2]. Disturbance or even breakdown of cerebral glucose metabolism and subsequent energy deficit is associated with several brain disorders, including acute stroke [1,3,4]. We recently reported the application and utility of Chemical Exchange Sensitive Spin Lock (CESL) magnetic resonance imaging (MRI) of the glucose analogue 2-deoxy-d-glucose (2DG) as a novel imaging biomarker to quantify glucose uptake and metabolism in the middle cerebral artery occlusion (MCAO) model of transient focal ischemic stroke in rats [5]. 2DG CESL MRI was compared to standard MRI imaging sequences measuring reduced cerebral blood flow (CBF) using perfusion MRI, and diffusion MRI of the apparent diffusion coefficient (ADC). ADC is a surrogate of the extent of the ischemic lesion core and the mismatch with perfusion MRI is a clinically established marker of the penumbra which is defined as potentially salvageable tissue when restoring perfusion [5]. In our study, we demonstrated that 2DG CESL MRI allowed measuring the cellular uptake and metabolism of the glucose analogue in ischemic tissue and allowed precise mapping of the hypometabolic ischemic core [5]. Importantly, 2DG CESL is not specific to brain ischemia. We believe that it can provide biomarkers of metabolism in many other animal models of brain disorders, such as brain tumors or neurodegenerative disease, or other models entirely.

The underlying principle of 2DG CESL MRI is based on measuring chemical exchange between exchangeable protons on the molecule and the bulk water pool, which can be performed for D-glucose and other glucose analogues as well [6–11]. In CESL, the relaxation rate in the rotating frame (R_1ρ_) is measured, which increases linearly with increasing concentration of 2DG protons in the non-water pool. Thus, the change ΔR_1ρ_ after 2DG injection compared to baseline is a marker of local 2DG concentration. The principle is very similar to measuring glucose via Chemical Exchange Saturation Transfer (gluco-CEST), but the sensitivity of CESL was shown to be higher [12].

2DG behaves chemically almost identical to 2-Deoxy-2-[^18^F]fluoroglucose (FDG), a well established tracer of metabolism in positron emission tomography (PET). Thus, 2DG CESL MRI may present an alternative to FDG-PET without the need of expensive radiochemistry and the high demands on logistics and legal administration of a radionuclide facility. Depending on the intended application, next to using 2DG CESL MRI, it might be useful considering replacing 2DG with the glucose analogue 3-O-methyl-D-glucose (3OMG) which is also detectable using CESL and contrary to 2DG is not metabolized by hexokinase [13].

**Fig 1:**
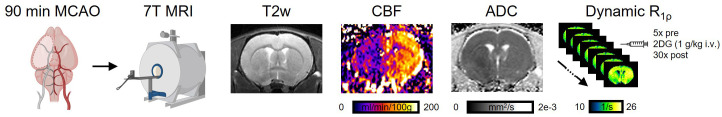
Overview of the protocol detailing experimental and analytical steps. **A)** Rats undergo 90 min transient MCAO. After surgery, animals are directly transferred to a 7 T MRI system for T2-weighted (T2w) MRI, perfusion MRI of cerebral blood flow (CBF), diffusion MRI of apparent diffusion coefficient (ADC) followed by dynamic R_1ρ_ mapping with CESL MRI before and after injection of 2DG. *Figure and legend were previously published and are reproduced from* [5] *under a CC-BY-NC license.*

Here, we provide an experimentally validated protocol [5] to measure cerebral uptake and metabolism of 2DG using CESL MRI in the context of stroke in rodents (Fig 1).

## Materials and methods

The protocol described in this peer-reviewed article is published on protocols.io, https://dx.doi.org/10.17504/protocols.io.n92ldnoz8v5b/v1 and is included for printing as supporting information S1 File with this article.

All animal procedures underlying the reporting of this protocol were performed after approval by the regulating authority (Landesamt für Gesundheit und Soziales Berlin). Studies were performed in accordance with the German Animal Welfare Act and EU regulations.

## Expected results

Using the protocol described herein, it will be possible to measure cerebral uptake and metabolism of the glucose analogue 2DG after MCAO as recently described [5]. Even though we expect that this protocol can be readily implemented, we suggest performing initial CESL measurements using phantoms of different 2DG concentrations similarly to outlined herein (supporting information S1 File).

**Fig 2:**
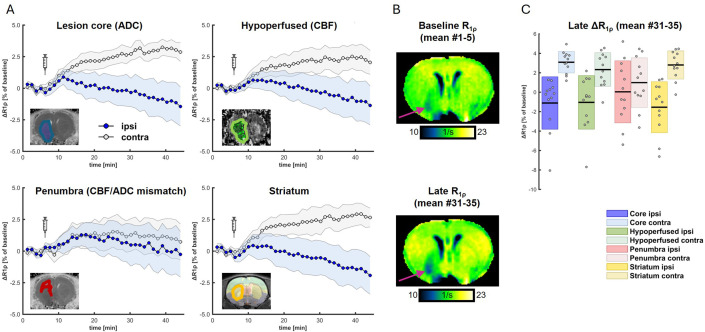
Example of in vivo metabolic MRI in the context of stroke. **A)** Quantification of mean R_1ρ_ in the lesion core, hypo-perfused areas, penumbra, ipsilateral striatum (blue) and in corresponding mirrored ROIs (gray) which showed strong differences continuously increasing over time. Shaded areas correspond to 95% confidence intervals (CI). Syringes indicate injection of 2DG at 8 minutes after start of CESL imaging. **B)** Representative baseline and late (mean of first and last 5 maps) R_1ρ_ maps show an increase in contralateral tissue and slight decrease in the lesion territory. **C)** Quantification of late R_1ρ_ showed strongest effects of ipsi- vs. contralateral values in striatum and lesion core. Here, the contrast was most pronounced in striatum (ipsi: −1.54 ± 2.62%, contra: 2.80 ± 1.45%, t = −5.27, p = 0.00026, significant after Bonferroni correction, Cohen’s d = 1.52) and lesion core (ipsi: −1.11 ± 2.70%, contra: 3.09 ± 1.10%, t = −5.08, p = 0.00036, significant after Bonferroni correction, Cohen’s d = 1.47) but smaller in hypoperfused tissue (ipsi: −1.04 ± 2.76%, contra: 2.33 ± 1.73%, t = −3.84, p = 0.0027, significant after Bonferroni correction, Cohen’s d = 1.11) and not significant in the penumbra (ipsi: 0.04 ± 3.19%, contra: 0.99 ± 2.55%, t = −1.16, p = 0.27, Cohen’s d = 0.33). *Figure and legend were previously published and are reproduced with modifications from* [5] *under a CC-BY-NC license.*

When performing 2DG CESL MRI in conjunction with standard measurements of blood flow and diffusion in the rat MCAO stroke model, it is expected that measurements follow previous results [5] showing an increase of R_1ρ_ over the period of CESL imaging in metabolically active tissue (e.g., contralateral to the stroke as shown here, Fig 2). However, R_1ρ_ is expected to be stable or to decrease in metabolically compromised tissue (e.g., in the stroke territory, Fig 2). Quantifications should focus on late ΔR_1ρ_ measurements, e.g., representing the mean of the last five scans (Fig 2C).

Our previous study focused on cerebral 2DG uptake and metabolism in stroke at the time of reperfusion [5]. However, with minimal adjustments, measurements can be made at any timepoint after reperfusion. Given the relatively high concentrations of 2DG required for this protocol, it might be advantageous to replace 2DG with other glucose analogues such as 3OMG to avoid 2DG toxicity from blocking hexokinase [14], which should be possible with minor modifications as performed in other studies [13]. Minor adjustments should allow the protocol to be adapted for use in other disease models and other organs.

In summary, here we have described a protocol for noninvasive metabolic imaging of the brain.

### Associated content

- Experimental study describing the utility of 2DG-CESL-MRI to measure cerebral glucose metabolism in a rat model of transient ischemic stroke: https://doi.org/10.1177/0271678X251355049
- Protocol on Protocols.io: https://doi.org/10.17504/protocols.io.n92ldnoz8v5b/v1
- Data and code associated with this manuscript are available on Zenodo (https://zenodo.org/records/14526092; DOI: 10.5281/zenodo.14526091), including MRI image data to reproduce the steps described in this protocol.

## Supporting information

S1 File Step-by-step protocol, also available on protocols.io (DOI: 10.17504/protocols.io.n92ldnoz8v5b/v1). (PDF)
