# Supplementary material for: Measuring cerebral glucose metabolism by chemical exchange-sensitive spin-lock (CESL) MRI of 2-deoxy-D-glucose in rodents
Source: PLoS One. 2026 Mar 27;21(3):e0346046. doi: 10.1371/journal.pone.0346046 (PMC13028544; doi:10.1371/journal.pone.0346046)
Supplement: S1 File — (PDF) [file pone.0346046.s001.pdf]

Mar 18, 2026

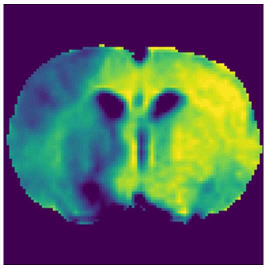

2-deoxy-D-glucose chemical exchange sensitive spin-lock (CESL) MRI of the rodent brain

DOI
https://dx.doi.org/10.17504/protocols.io.n92ldnoz8v5b/v1

Philipp Boehm-Sturm[1,2,3], Patrick Schuenke[4,5], Marco Foddis[1,2,3], Susanne Mueller[1,2,3], Stefan P Koch[1,2,3], Daniel J Beard[6], Paul Holloway[7,8], Amin Mottahedin[7,8,9], Leif Schröder[10,11,12], Alastair M Buchan[1,7,8], Philipp Mergenthaler[1,3,7,8]

[1]Charité - Universitätsmedizin Berlin, Center for Stroke Research Berlin, Charitéplatz 1, 10117 Berlin, Germany;
[2]Charité - Universitätsmedizin Berlin, Experimental Imaging at the Charité for 3R (EPIC3R), Charité 3R – Replace | Reduce | Refine, Berlin, Germany;
[3]Charité - Universitätsmedizin Berlin, Department of Neurology with Experimental Neurology, Berlin, Germany;
[4]Charité - Universitätsmedizin Berlin, Department of Radiology, Berlin, Germany;
[5]Physikalisch-Technische Bundesanstalt (PTB), Braunschweig and Berlin, Germany;
[6]School of Biomedical Sciences and Pharmacy, The University of Newcastle, Newcastle, Australia;
[7]Radcliffe Department of Medicine, University of Oxford, Oxford, UK;
[8]Consortium International pour la Recherche Circadienne sur l'AVC (CIRCA);
[9]Nuffield Department of Clinical Neurosciences, University of Oxford, Oxford, UK;
[10]German Cancer Research Center, Translational Molecular Imaging, Heidelberg, Germany;
[11]Department for Physics and Astronomy, Ruprecht Karls University Heidelberg, Heidelberg, Germany;
[12]German Cancer Consortium (DKTK), Heidelberg, Germany

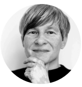

Susanne Mueller
Charité Universitätsmedizin Berlin

Create & collaborate more with a free account

Edit and publish protocols, collaborate in communities, share insights through comments, and track progress with run records.

Create free account

OPEN ACCESS

DOI: https://dx.doi.org/10.17504/protocols.io.n92ldnoz8v5b/v1

External link: https://doi.org/10.1177/0271678X251355049

Protocol Citation: Philipp Boehm-Sturm, Patrick Schuenke, Marco Foddis, Susanne Mueller, Stefan P Koch, Daniel J Beard, Paul Holloway, Amin Mottahedin, Leif Schröder, Alastair M Buchan, Philipp Mergenthaler 2026. 2-deoxy-D-glucose chemical exchange sensitive spin-lock (CESL) MRI of the rodent brain. **protocols.io**
https://dx.doi.org/10.17504/protocols.io.n92ldnoz8v5b/v1

Manuscript citation:
Boehm-Sturm P, Schuenke P, Foddis M, Mueller S, Koch SP, Beard DJ, Holloway P, Mottahedin A, Schröder L, Buchan AM, Mergenthaler P. 2-deoxy-D-glucose chemical exchange-sensitive spin-lock MRI of cerebral glucose metabolism after transient focal stroke in the rat. J Cereb Blood Flow Metab. 2025 Jul 8:271678X251355049. doi: 10.1177/0271678X251355049.
https://doi.org/10.1177/0271678X251355049

License: This is an open access protocol distributed under the terms of the **Creative Commons Attribution License**, which permits unrestricted use, distribution, and reproduction in any medium, provided the original author and source are credited

Protocol status: Working
We use this protocol and it's working.

Created: June 23, 2025

Last Modified: March 18, 2026

Protocol Integer ID: 220711

Keywords: MRI, stroke, MCAO, CESL, Glucose Metabolism, 2-deoxy-D-glucose, metabolic MRI, metabolic brain imaging, brain metabolism, mri with the glucose analogue, glucose chemical exchange sensitive spin, mri of the rodent brain, sensitive to various glucose analog, various glucose analog, glucose analogue, glucose metabolism, rat model of stroke, magnetic resonance imaging, metabolic changes in ischemic tissue, glucose, mri, glucose chemical exchange, sensitive spin, rodent brain, therapeutic responses in neurological condition, rat brain, metabolic change, ischemic tissue, cesl mri, monitoring therapeutic response

Funders Acknowledgements:
Einstein Foundation Berlin
Grant ID: EJF-2020-602
Einstein Foundation Berlin
Grant ID: EVF-2021-619
Einstein Foundation Berlin
Grant ID: EVF-2021-619-2
Leducq Foundation for Cardiovascular and Neurovascular Research
Grant ID: 21CVD04
Einstein Foundation Berlin
Grant ID: EVF-BUA-2022-694

Abstract

Magnetic resonance imaging (MRI) of glucose metabolism shows significant potential for identifying disease biomarkers and monitoring therapeutic responses in neurological conditions. Here, we present a protocol utilizing chemical exchange-sensitive spin-lock (CESL) MRI with the glucose analogue 2-deoxy-D-glucose (2DG) in the rat brain. We employed this method to characterize metabolic changes in ischemic tissue within a rat model of stroke. However, the technique is not limited to stroke and may be adapted to other disease models with minimal modifications. Previous research has demonstrated that CESL MRI is sensitive to various glucose analogs, including regular D-glucose, which is suitable for human application. Consequently, our protocol provides a foundation for a wide range of future applications in both basic and translational research, with potential utility in animal models and, eventually, human studies.

Image Attribution

Protocol title image is reproduced under a CC-BY license from *Boehm-Sturm P, Schuenke P, Foddis M, Mueller S, Koch SP, Beard DJ, Holloway P, Mottahedin A, Schröder L, Buchan AM, Mergenthaler P. Open Data Repository - 2-deoxy-D-glucose chemical exchange-sensitive spin-lock MRI of cerebral metabolism after stroke in the rat. Zenodo. [Data set] doi: 10.5281/zenodo.14526091.* https://doi.org/10.5281/zenodo.14526091.

Materials

Bruker 7 Tesla BioSpec (70/20 USR) with Avance III HD Electronics and Paravision 6.0.1 Software
Transmit-only Volume Coil for Mice and Rats - 86mm (Bruker, Ettlingen, Germany)
Phased Array Surface Coil for Rat Head (Rapid Biomed, Rimpar, Germany)

Small Animal Monitoring System (SA Instruments, Stony Brook, NY, USA)

Femoralis catheter: Portex Fine Bore Polythene 30 Tubing, 0.58 mm inner diameter, 0.96 mm outer diameter, 90 cm long

Filament for MCAO: 390 µm diameter, 4039910PK10Re, Doccol, Sharon, 5 MA, USA

2-Desoxy-D-Glucose (Carl Roth, Art.-Nr. CN96.3; Germany)

Matlab Toolbox ANTx2 https://github.com/ChariteExpMri/antx2

Protocol materials

- 2-Desoxy-D-Glucose **Carl Roth Catalog #**CN96.3
- Gibco DPBS without Calcium and Magnesium **Fisher Scientific Catalog #**14190136 or equivalent

Troubleshooting

Animal preparation

1 Animals undergo two surgical procedures before the MRI examination:
a. Femoralis catheter implantation to inject 2DG during the MRI measurements.
b. 90 min middle cerebral artery occlusion (MCAO) to induce focal cerebral ischemia.

> Note
>
> All animal procedures mentioned herein are specific to stroke modeling in the rat using 90 min MCAO and can be replaced with other disease models.

2 Anesthetize animals using isoflurane in a 70%/30% N2O/O2 mixture. Anesthesia is induced with 5% isoflurane and maintained at 1.75-2.5% isoflurane during the femoralis catheter placement and the MCAO and 1.75% during MRI to achieve a breathing rate of ~60-100/min.

3 For MRI, place the animal on a heated water blanket to ensure constant body temperature of 37±0.5 °C.

4 Use ear and toothbar fixation to minimize motion artifacts.

5 Monitor the respiration rate using a small animal monitoring system (SA Instruments, Stony Brook, NY, USA).

Preparation of 2-deoxy-D-glucose (2DG) solution

6 Goal: injected dose of 1 g/kg body weight.

6.1 Reagent: 2-Desoxy-D-Glucose **Carl Roth Catalog** #CN96.3

6.2 Preparation of the injection solution: 25% 2DG w/v, i.e. 0.25 g in 1 mL of solution.
~3.3 mL NaCl and 1 g 2DG were needed to achieve 4 mL of the final solution.

6.3 Injection volume: v = dose * weight / concentration = 1 g/kg * weight / 0.25g / mL = 4 mL/kg.
Example: 1.6 mL for a rat of 400 g body weight.

6.4 Injection period: around 1-2 min.

MRI hardware

7

Equipment	
BioSpec	NAME
70/20 USR	TYPE
Bruker	BRAND
https://www.bruker.com/	LINK

Bruker 7 Tesla system (BioSpec 70/20 USR, Bruker, Ettlingen, Germany) + 86 mm transmit volume resonator (Bruker) + receive rat brain surface coil (Bruker). Paravision 6.0.1 software.

MRI pulse sequences

8 The MRI protocol includes the following scan protocols (Boehm-Sturm, et al., 2025).

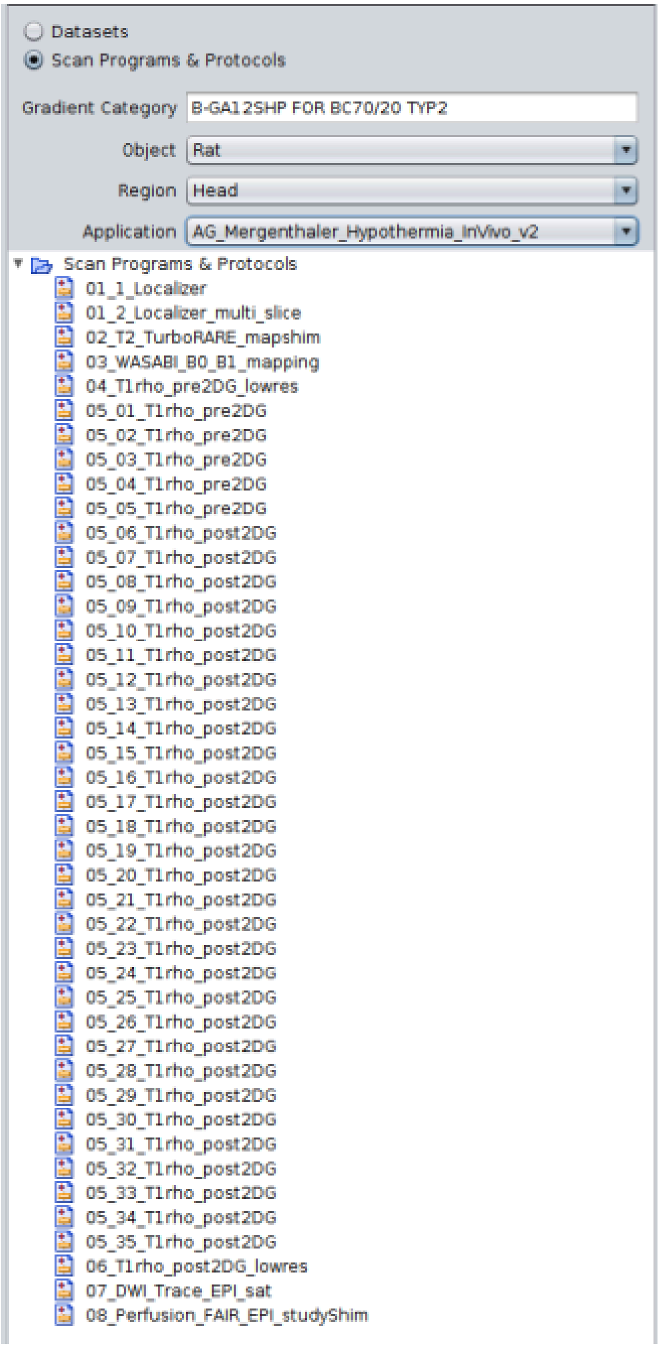

Glossary:

- RARE: rapid acquisition with relaxation enhancement (turbo spin echo sequence)
- WASABI: Simultaneous mapping of water shift and B1 (Schuenke P, Windschuh J, et al., 2017)
- T1rho: CESL sequence to map R1rho=1/T1rho
- DWI: diffusion weighted imaging
- FAIR: flow sensitive alternating inversion recovery (sequence for perfusion imaging)
- EPI: echo planar imaging (DWI and FAIR use spin echo EPI readout)

In vivo MRI protocol

9 **01_1_Localizer**

Note

When the animal is placed into the scanner first tune and match (wobble) the volume resonator.

Run scan with Automatic Setup / Adjustments.

10 **01_2_Localizer_multi_slice**

Run scan.

11 **02_T2_TurboRARE_mapshim**

11.1 **Set Geometry**

a. Reduce the number of slices to 6, adjust the slice position so that the anterior commissure is covered by the uppermost slice.
b. Switch back to 38 slices and adjust center to trachea.
c. Adjust the ellipsoid shim volume to the center of the brain.
d. Make sure that the **read offset is 0.00.**

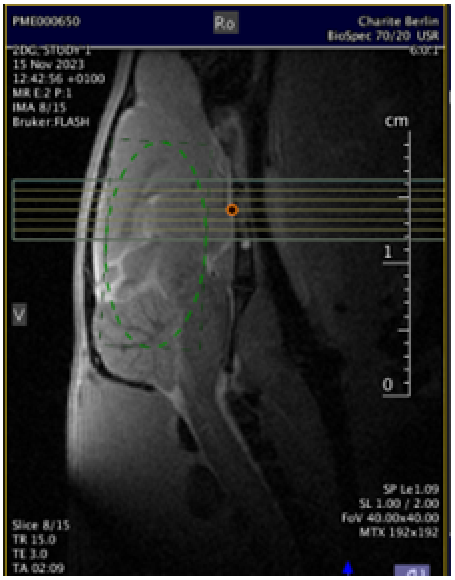

a. slice positioning

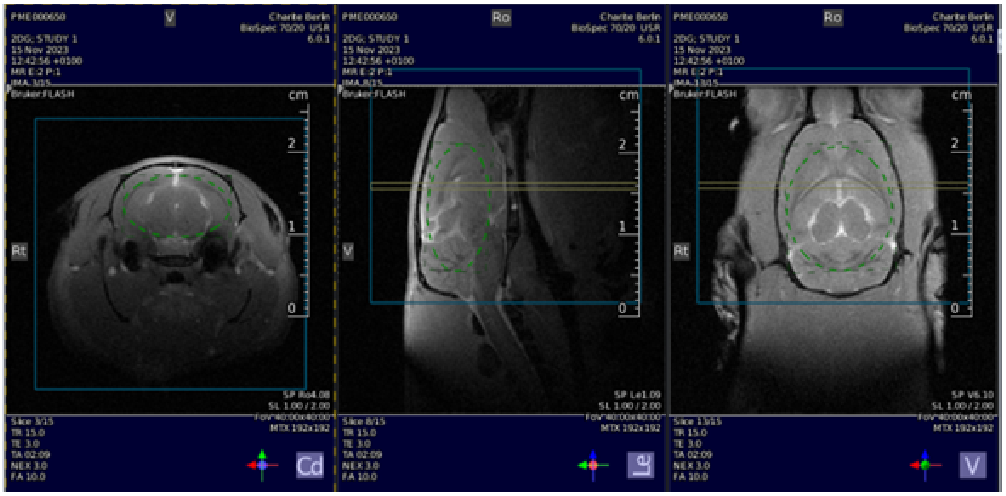

b. /c. final geometry and shim volume position

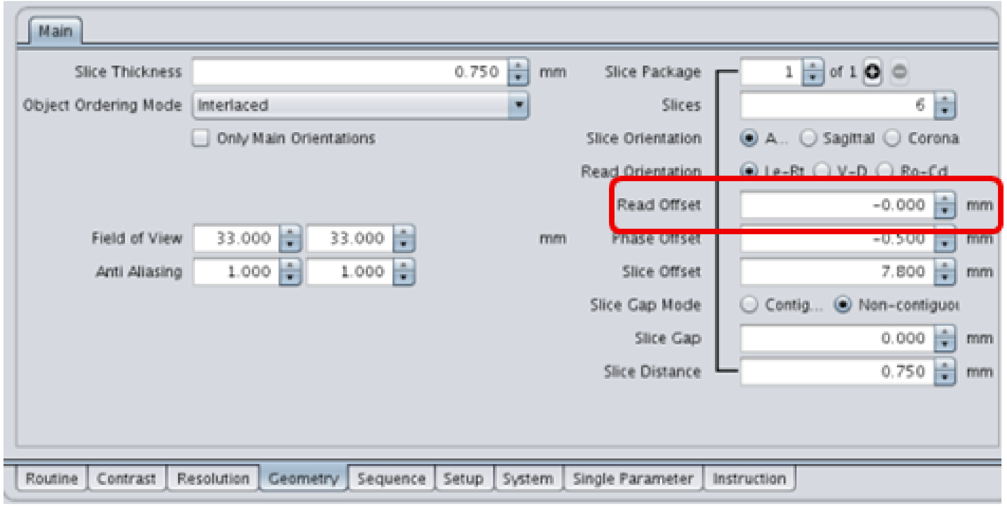

d. check read offset

11.2 **B0 map**

Create a B0 map with:

a. 3 averages, default FOV and

b. Map S/N 20.

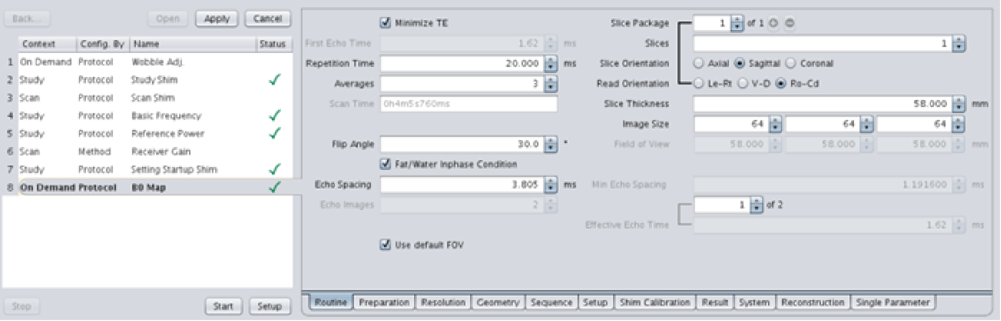

a. B0 map settings FOV and averages

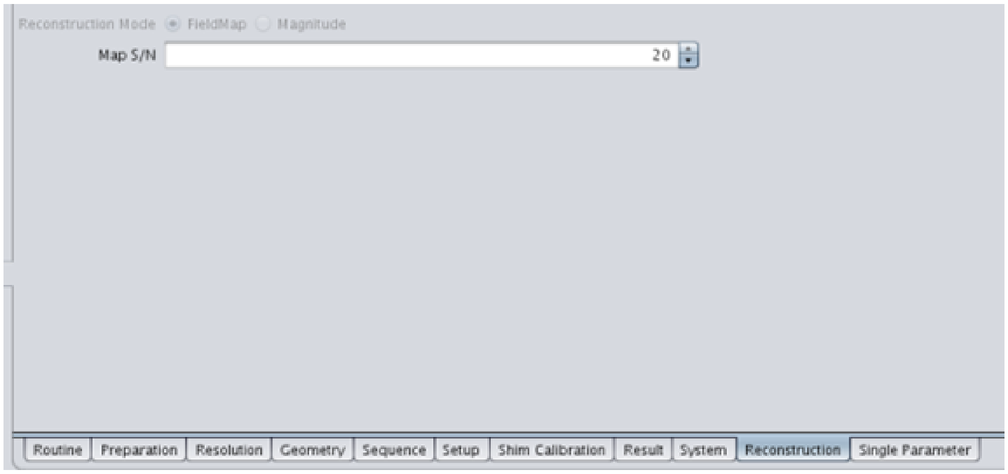

b. B0 map setting Reconstruction

Run B0 mapping (Start).

11.3 Run scan 02_T2_TurboRARE_mapshim.

12 **03_WASABI_B0_B1_mapping**

Import the slice orientation of 02_T2_TurboRARE_mapshim and add 2.625 mm to the slice offset!

Note

B0 and B1 mapping is recommended to check B0/B1 homogeneity in the given experimental setup and to correct for inhomogeneities in the image post processing step.
In our experimental setting, the B0 and B1 maps were only used once to check sufficient homogeneity and not used for corrections.
WASABI is particularly efficient in mapping B0 and B1 but other sequences can be used.

Note

The slice offset is specific to the MCAO model to ensure the center of the striatum is covered, which is the brain region most severely affected by the stroke. In other disease models different offsets can be used to perform 2DG-CESL MRI in the brain regions of interest.

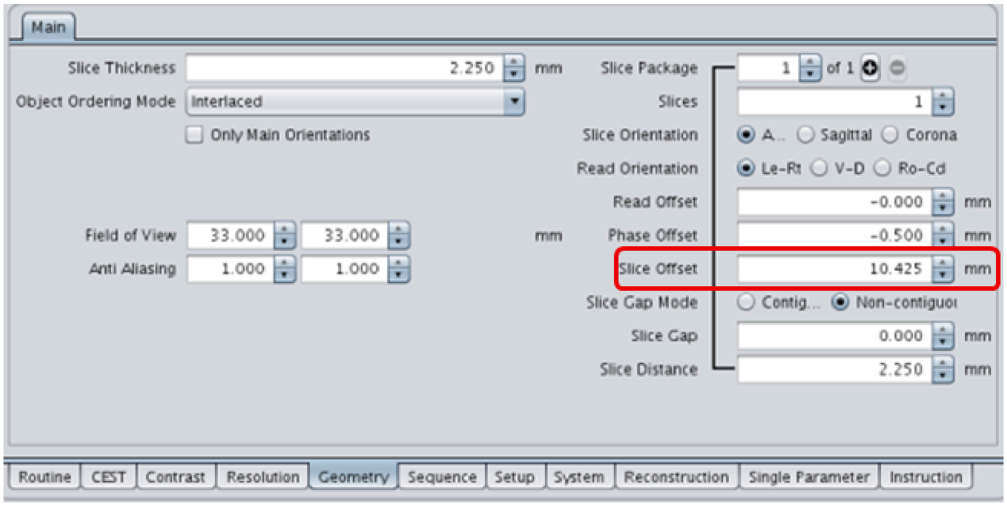

a. set the slice offset

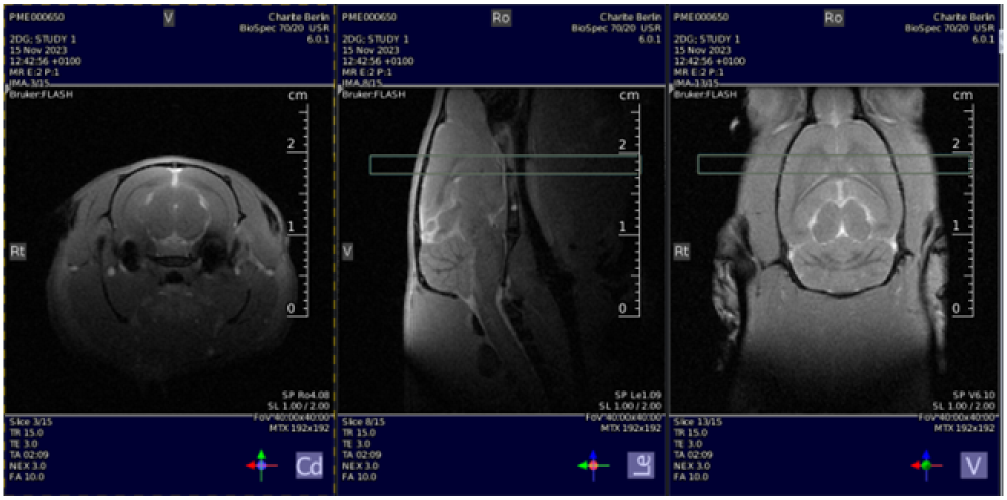

b. final geometry to cover the center of the striatum

Run scan.

13 **Import the slice orientation of 03_WASABI_B0_B1_mapping to all following T1rho protocols**

(04_T1rho_pre to 06_T1rho_post)

14 **04_T1rho_pre2DG_lowres**

05_1_T1rho_pre2DG to 05_5_Tirho_pre2DG

Run scans.

15 **⇛ Slow injection of 2DG solution through the femoralis catheter over 2 min!**

Inject the calculated volume plus the dead volume of the catheter.

16 **05_6_T1rho_post2DG to 05_35_T1rho_post2DG**

Run scans.

17 **06_T1rho_post2DG_lowres**

Run scan.

18 **07_DWI_Trace_EPI_sat**

Note

Diffusion (DWI) and perfusion (PWI) MRI are of specific interest in the stroke model. These sequences can also be of interest in other disease contexts but are not mandatory for CESL imaging.

Import the slice orientation of 02_T2_TurboRARE_mapshim and adjust the FOV Saturation slice.

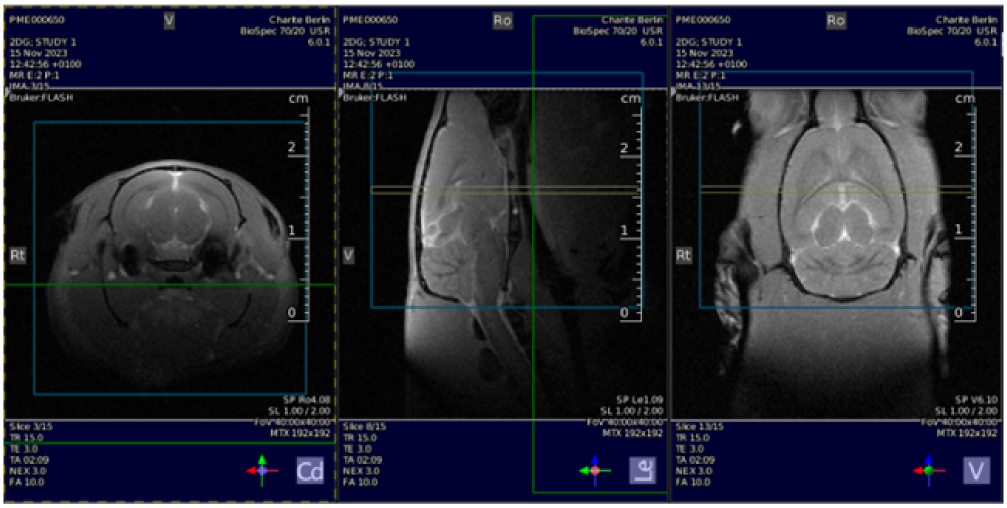

final geometry with FOV saturation slice

Run scan.

19 **08_Perfusion_FAIR_EPI_studyshim**

a. Import the slice orientation of 03_WASABI scan.

b. Use the **study shim** option. Use of a global shim ensures a better global inversion pulse of the FAIR sequence.

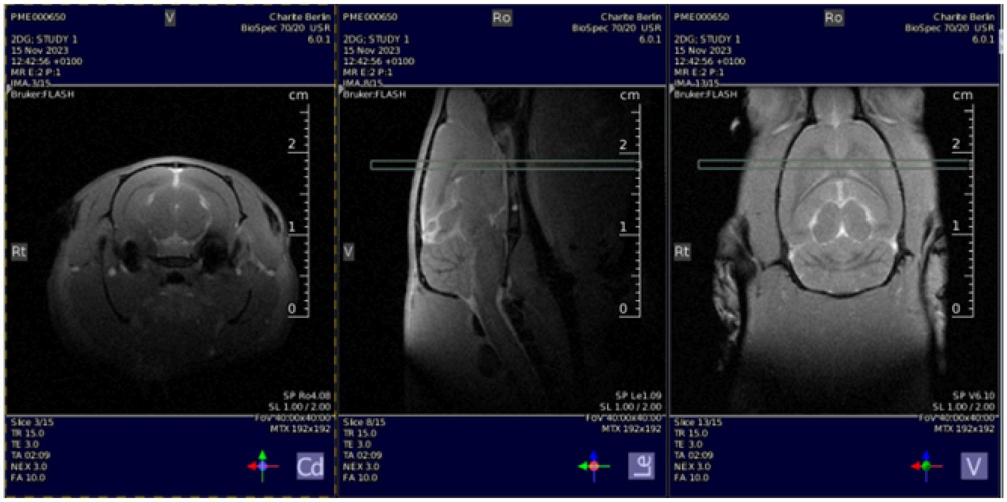

a. final geometry

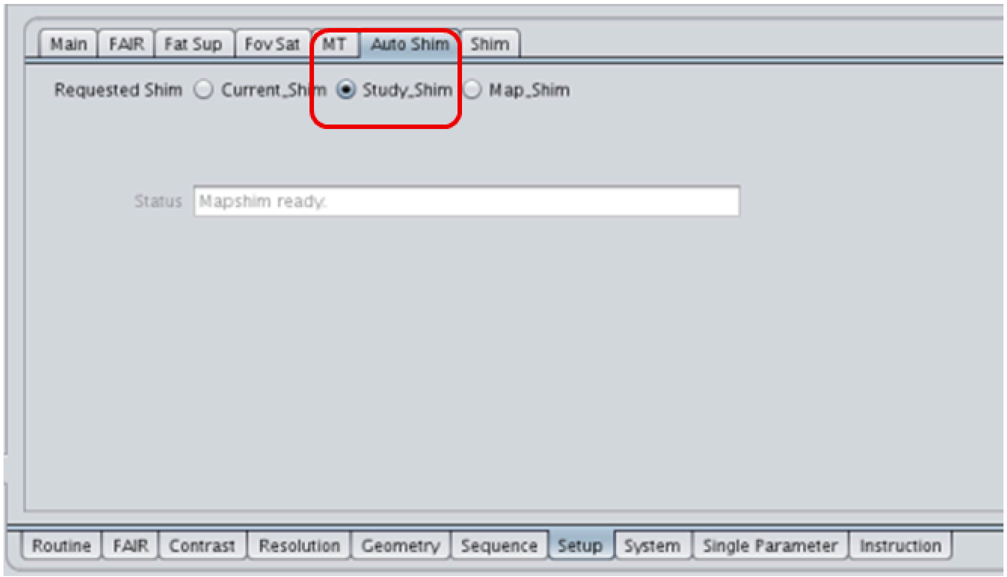

b. check study shim option

Run scan.

Image data processing

20 **Generate $R_{1\rho}$ maps**

Calculate $R_{1\rho}(t_i)$ at time t_i voxelwise via monexponential fitting of the signal over the spin lock times TSL:

$$S\left(TSL, t_i\right) = e^{-R_{1\rho}(t_i)\cdot \mathrm{TSL}};\ i = 1 \ldots 35$$

21 **Region of Interest (ROI) Analysis**

ROI analysis can be performed in two ways:

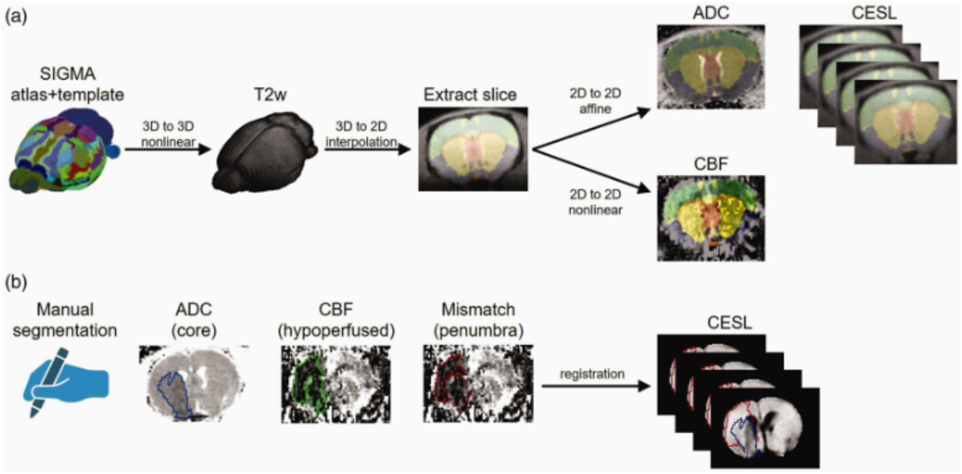

ROI analyses. CESL images can be quantified in anatomical regions defined in the SIGMA rat brain atlas (a) or using manual delineation (b) of the lesion on ADC, CBF and late $\Delta R_{1\rho}$ maps (mean of the last 5 maps). Penumbra is defined via perfusion/diffusion mismatch.
Figure reproduced from Boehm-Sturm et al., 2025 (doi: 10.1177/0271678X251355049) under a CC-BY-NC license.

21.1 **Automatic analysis of brain regions of interest**

Segment brain regions automatically on the different MRI contrasts (T2, ADC, CBF, CESL) using a series of 3D to 3D and 3D to 2D image registrations using the SIGMA

rat brain template (Barriere, et al., 2019). The registration can be performed for example in ANTx2 software (Koch, et al., 2019).

Software	
ANTx2	NAME
all all	OS
ChariteExpMRI	DEVELOPER
https://github.com/ChariteExpMri/antx2	REPOSITORY
https://github.com/ChariteExpMri/antx2	SOURCE LINK

Apply the registration to the SIGMA atlas, which matches the template. For registration, use the b = 0 image for diffusion MR images, the first inversion time image for perfusion MR images and the first TSL image for CESL images. For perfusion MRI, images will be distorted due to shimming artefacts of the EPI sequence and a nonlinear registration is needed. For ADC and CESL images, affine (12 degrees of freedom) registrations are sufficient.

21.2 Manual analysis of stroke regions of interest

Note

This step is only executed for the stroke model. It could be of interest for other animal models to segment special regions of interest manually but is not mandatory.

Segment the hypointense lesion on ADC, CBF and $\Delta R_{1\rho}$ maps (mean image of the last 5 images) using an initial automated histogram-based segmentation followed by manual corrections in ANALYZE software (v5.0, AnalyzeDirect, Overland Park, KS, USA). Using the image registration transforms of the first approach, transform the lesion masks from ADC and CBF to the atlas and then register them to the $\Delta R_{1\rho}$ maps. In atlas space, generate ROIs mirrored at the midline and transform them to the $\Delta R_{1\rho}$ maps. Define lesion core via hypointense ADC, hypoperfused tissue via hypointense CBF and the hypometabolic lesion via hypointense areas on the mean $\Delta R_{1\rho}$ maps (mean of the last 5 measurements). The penumbra is defined via perfusion diffusion mismatch, i.e.

$$mask_{penumbra} = mask_{hypoperfused} \setminus \left(mask_{hypoperfused} \bigcap mask_{core} \right)$$

22 **Statistics**

Extract mean values from regions of interest. Perform group statistical analyses on those values with tests matching the experimental design of your study.

In vitro quality assurance

23 Prepare phantom with 5 mm NMR tubes containing different concentrations of 2DG in buffer. Typical range of concentrations: (0, 0.02 M, 0.04 M, 0.06 M, 0.08 M, 0.10 M) 2DG in DPBS:

Gibco DPBS without Calcium and Magnesium **Fisher Scientific Catalog #**14190136 or equivalent

24 Perform the in vivo CESL MRI protocol excluding the DWI and PWI scans. A single T1rho scan is sufficient. Use a single slice perpendicular to the NMR tubes (cf. T1rho protocol in section "In vivo MRI").

25 Generate $R_{1\rho}$ map (cf. section "Image data processing").

26 Plot $R_{1\rho}$ over 2DG concentration and perform a linear fit. The result should be similar to this

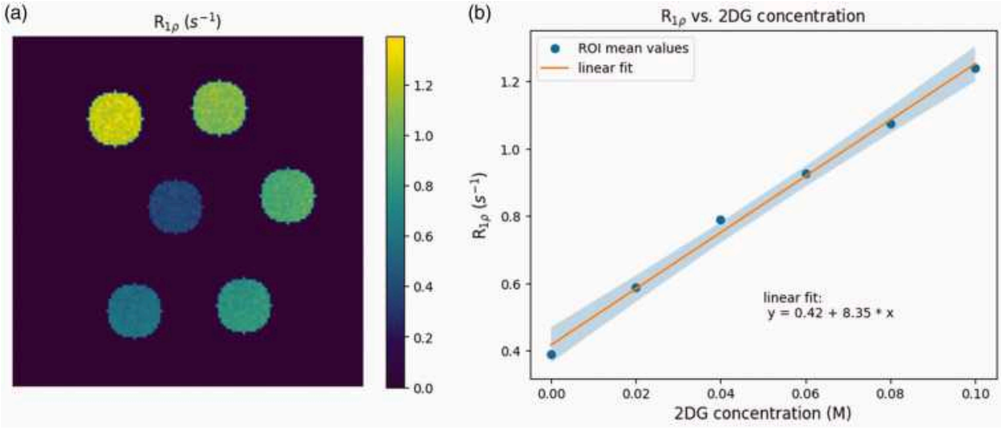

CESL MRI of tubes containing different concentrations of 2DG in DPBS. $R_{1\rho}$ map (a) and quantification including linear fit (b).
*Figure reproduced from Boehm-Sturm et al., 2025 (doi: **10.1177/0271678X251355049**) under a CC-BY-NC license.*

Protocol references

Boehm-Sturm P, Schuenke P, Foddis M, Mueller S, Koch SP, Beard DJ, Holloway P, Mottahedin A, Schröder L, Buchan AM, Mergenthaler P. 2-deoxy-D-glucose chemical exchange-sensitive spin-lock MRI of cerebral glucose metabolism after transient focal stroke in the rat. *J Cereb Blood Flow Metab* 2025; 271678X251355049. DOI: **10.1177/0271678X251355049**.

Boehm-Sturm P, Schuenke P, Foddis M, Mueller S, Koch SP, Beard DJ, Holloway P, Mottahedin A, Schröder L, Buchan AM, Mergenthaler P. Open Data Repository - 2-deoxy-D-glucose chemical exchange-sensitive spin-lock MRI of cerebral metabolism after stroke in the rat. [Data set]. *Zenodo* 2024; DOI: **10.5281/zenodo.14526091**.

Barriere DA, Magalhaes R, Novais A, Marques P, Selingue E, Geffroy F, Marques F, Cerqueira J, Sousa JC, Boumezbeur F, Bottlaender M, Jay TM, Cachia A, Sousa N, Meriaux S. The SIGMA rat brain templates and atlases for multimodal MRI data analysis and visualization. *Nat Commun* 2019; 10: 5699. DOI: **10.1038/s41467-019-13575-7**.

Herz K, Lindig T, Deshmane A, Schittenhelm J, Skardelly M, Bender B, Ernemann U, Scheffler K, Zaiss M. T1rho-based dynamic glucose-enhanced (DGErho) MRI at 3 T: method development and early clinical experience in the human brain. *Magn Reson Med* 2019; 82: 1832–1847. DOI: **10.1002/mrm.27857**.

Jin T, Kim SG. Advantages of chemical exchange-sensitive spin-lock (CESL) over chemical exchange saturation transfer (CEST) for hydroxyl- and amine-water proton exchange studies. *NMR Biomed* 2014; 27: 1313–1324. DOI: **10.1002/nbm.3191**.

Jin T, Mehrens H, Hendrich KS, Kim SG. Mapping brain glucose uptake with chemical exchange-sensitive spin-lock magnetic resonance imaging. *J Cereb Blood Flow Metab* 2014; 34: 1402–1410. DOI: **10.1038/jcbfm.2014.97**.

Jin T, Iordanova B, Hitchens TK, Modo M, Wang P, Mehrens H, Kim SG. Chemical exchange-sensitive spin-lock (CESL) MRI of glucose and analogs in brain tumors. *Magn Reson Med* 2018; 80: 488–495. DOI: **10.1002/mrm.27183**.

Jin T, Mehrens H, Wang P and Kim SG. Chemical exchange-sensitive spin-lock MRI of glucose analog 3-O-methyl-d-glucose in normal and ischemic brain. *J Cereb Blood Flow Metab* 2018; 38(5): 869–880. DOI: **10.1177/0271678X17707419**.

Koch S, Mueller S, Foddis M, Bienert T, von Elverfeldt D, Knab F, Farr TD, Bernard R, Dopatka M, Rex A, Dirnagl U, Harms C, Boehm-Sturm P. Atlas registration for edema-corrected MRI lesion volume in mouse stroke models. *J Cereb Blood Flow Metab* 2019; 39: 313–323. DOI: **10.1177/0271678X17726635**.

Paech D, Schuenke P, Koehler C, Windschuh J, Mundiyanapurath S, Bickelhaupt S, Bonekamp D, Baumer P, Bachert P, Ladd ME, Bendszus M, Wick W, Unterberg A, Schlemmer HP, Zaiss M, Radbruch A. T1rho-weighted dynamic glucose-enhanced MR imaging in the human brain. *Radiology* 2017; 285: 914–922.DOI: **10.1148/radiol.2017162351**.

Schuenke P, Koehler C, Korzowski A, Windschuh J, Bachert P, Ladd ME, Mundiyanapurath S, Paech D, Bickelhaupt S, Bonekamp D, Schlemmer HP, Radbruch A, Zaiss M. Adiabatically prepared spin-lock approach for T1rho-based dynamic glucose enhanced MRI at ultrahigh fields. *Magn Reson Med* 2017; 78: 215–225. DOI: **10.1002/mrm.26370**.

Schuenke P, Paech D, Koehler C, Windschuh J, Bachert P, Ladd ME, Schlemmer HP, Radbruch A, Zaiss M. Fast and quantitative T1rho-weighted dynamic glucose enhanced MRI. *Sci Rep* 2017; 7: 42093.
DOI: **10.1038/srep42093**.

Schuenke P, Windschuh J, Roeloffs V, Ladd ME, Bachert P, Zaiss M. Simultaneous mapping of water shift and B_1 (WASABI)-Application to field-Inhomogeneity correction of CEST MRI data. *Magn Reson Med*. 2017; 77(2):571-580. doi: **10.1002/mrm.26133**.

Xu X, Yadav NN, Knutsson L, Hua J, Kalyani R, Hall E, Laterra J, Blakeley J, Strowd R, Pomper M, Barker P, Chan K, Liu G, McMahon MT, Stevens RD, van Zijl PC. Dynamic Glucose-Enhanced (DGE) MRI: translation to human scanning and first results in glioma patients. *Tomography* 2015; 1: 105–114. DOI: **10.18383/j.tom.2015.00175**.

Acknowledgements

This work was funded by the Einstein Foundation Berlin (EJF-2020-602 to PM, EVF-2021-619 and EVF-2021-619-2 to PM and AMB) and the Leducq Foundation for Cardiovascular and Neurovascular Research (Leducq Foundation Trans-Atlantic Network of Excellence on Circadian Effects in Stroke, 21CVD04, PM and AMB). Funding to SM, MF, SPK and PBS was provided by the German Federal Ministry of Education and Research (BMBF) under the ERA-NET NEURON scheme (01EW2305), and the DFG (project BO 4484/2-1, Project-ID 424778381-TRR 295 ReTune and EXC-2049-390688087 NeuroCure). Noninvasive imaging experiments were supported by Charité 3R – Replace | Reduce | Refine. Funding to PS was provided by the DFG (Project-ID 372486779-SFB 1340 Matrix in Vision). DJB was supported by an EMBO short-term Fellowship and funding from the National Health and Medical Research Council Australia (APP1182153). PM is Einstein Junior Fellow and AMB is Einstein Visiting Fellow, both funded by the Einstein Foundation Berlin. PM acknowledges funding from the Einstein Foundation Berlin (EVF-BUA-2022-694), the Volkswagen Foundation (9A866), the Else Kröner-Fresenius Stiftung (2019-A34), and the Stiftung Charité (StC-VF-2024-59). Besides funding, the sponsoring organizations did not play any role in the preparation, review, or approval of the article, or decision to submit the article for publication.
